# Perspectives, past, present and future: the proline cycle/proline-collagen regulatory axis

**DOI:** 10.1007/s00726-021-03103-7

**Published:** 2021-11-26

**Authors:** James M. Phang

**Affiliations:** Scientist Emeritus, Mouse Cancer Genetics Program, CCR, NCI at Frederick, National Institutes of Health, Frederick, MD 21702 USA

**Keywords:** Metabolic reprogramming, Cancer therapy, Redox, Extracellular matrix, Nucleotides

## Abstract

In the 35 years since the introduction of the “proline cycle”, its relevance to human tumors has been widely established. These connections are based on a variety of mechanisms discovered by many laboratories and have stimulated the search for small molecule inhibitors to treat cancer or metastases. In addition, the multi-layered connections of the proline cycle and the role of proline and hydroxyproline in collagen provide an important regulatory link between the extracellular matrix and metabolism.

## Introduction

The first review of proline’s regulatory effects on cell metabolism appeared 35 years ago (Phang 1985), but accelerating discoveries during the last 10 years established the central role of proline in cancer biology. Supporting evidence has come from: unbiased metabolomics (Tang et al. 2018), dysregulation of proline enzymes associated with cancer (D’Aniello et al. 2020), maintenance or disruption of redox homeostasis (Schworer et al. 2020); signaling mechanisms for cell growth pathways (mTOR and AAR-ATF4) (D'Aniello et al. 2015), tumor suppressors (P53) (Donald et al. 2001), and oncogenes (MYC, PI3K-AKT) (Tang et al. 2018; Liu et al. 2015). The linkage of proline metabolism is not limited to cancer but is also evident in viral proliferation as seen in the hijacking of proline biosynthetic enzymes (PYCR1) by Kaposi’s sarcoma herpes virus K1 (Choi et al. 2020) and MYC-dependent adenoviral proliferation (Thai et al. 2015). These exciting findings suggest that proline metabolism is a critical system for integrating the signals in cancer and/or viral metabolic reprogramming and that pharmacologic targeting of proline metabolism may provide novel therapeutic strategies not only for cancer (Tanner et al. 2018) but also for other diseases.

How did the interest in proline metabolism begin and what stimulated these aforementioned discoveries? Genomics provided ground-breaking approaches to the discoveries in carcinogenesis, but it was soon obvious that epigenetics initiated by metabolism and the microenvironment were critical. In another context, biochemists recognized that proline is the sole proteinogenic secondary (imino) acid and uniquely influences protein structure (Adams 1970). There were no suggestions, however, that proline metabolism would be the source of oncogenic properties. The initial studies on oncometabolism emphasized the diversion of glucose to lactate via glycolysis thereby conserving carbon moieties for cellular building blocks rather than consuming it to CO_2_ by oxidative phosphorylation (Vander Heiden et al. 2012); the nonessential amino acids were not considered important with the exception of glutamine which is essential for de novo nucleotide synthesis and serves as anaplerotic source for the TCA cycle (Altman et al. 2016). Thus, for the historical record, a brief narrative on the development of research on proline metabolism may be of interest.

Although the enzymes of proline metabolism had been identified and characterized by the 1970s (Adams 1970), there were few studies emphasizing their role in human disorders. The lack of sensitive methodology for cell studies was an obstacle. Radioisotopically labeled pyrroline-5-carboxylate (P5C) was enzymatically produced from precursor ornithine with partially purified rat liver ornithine aminotransferase (Smith et al. 1977). This served as a substrate for a sensitive method for P5C Reductase (PYCR) (Valle et al. 1973) and P5C Dehydrogenase (P5CDH) (Valle et al. 1974). Proline dehydrogenase (PRODH) (Phang et al. 1975) and P5C synthetase (Smith et al. 1980) assays were developed using similar strategies using commercially available uniformly radiolabeled substrates. Later, chemically synthesized DL-P5C was available from commercial sources. Using a similar approach, an assay for P5C synthetase was developed (Smith et al. 1980).

**Fig. 1:**
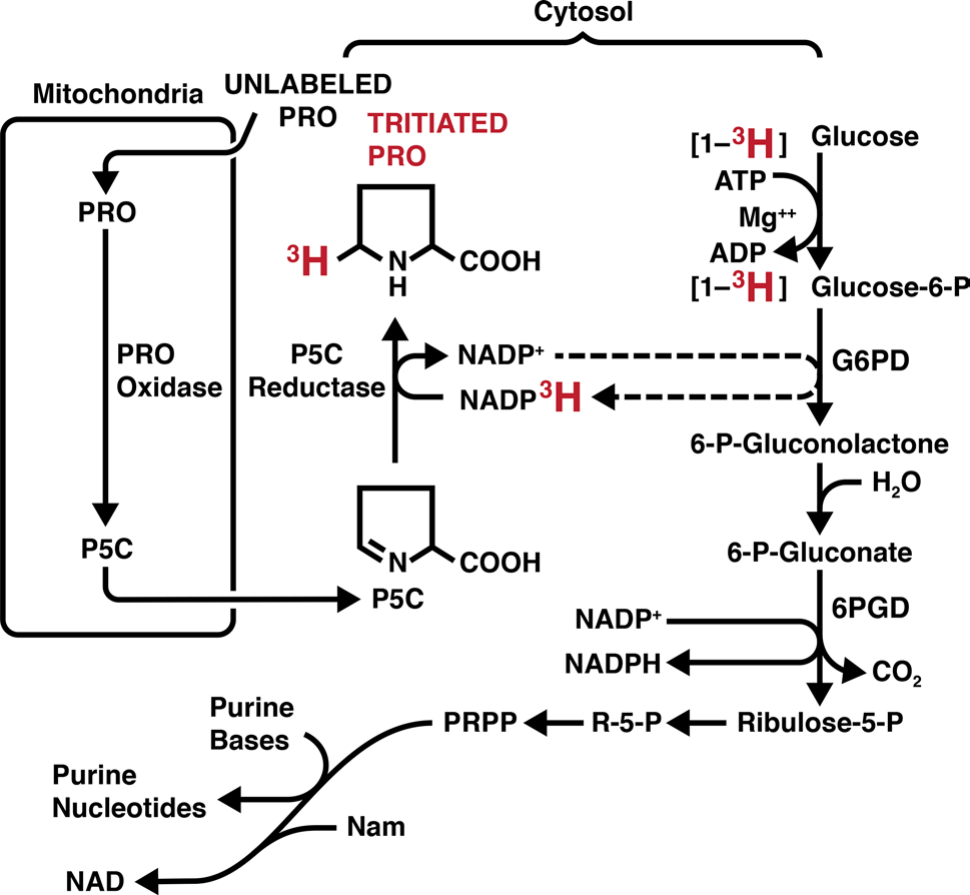
Experimental evidence supporting the “proline-P5C Cycle” (Hagedorn and Phang 1983). Using preparations of isolated rat liver mitochondria and dialyzed supernatants of sonicated human erythrocytes, we incubated [1-^3^H] glucose and monitored the production of [^3^H]-proline. Unlabeled proline, NADP^+^, ATP and rotenone were included. If the amount of triated proline produced in the complete incubation system is defined as 1.000, the following were obtained when a specific reactant was omitted. Minus rotenone, 0 0.055; minus proline, 0.009; minus NADP^+^, 0.003; minus mitochondria, 0.011; minus erythrocyte extract, 0.003. Tracing the tritium from [1-^3^H]-glucose, it is transferred to NADP^+^ to P5C produced from unlabeled proline to yield tritiated proline. No tritiated proline is formed ini the absence of NADP^+^ or unlabeled proline. In another study added P5C markedly increased the activity of the oxidative pentose phosphate pathway as well as the levels of R-5-P and PRPP. (Yeh et al. 1981) In a separate publication, we showed that in red cells with G6PD deficiency, the P5C-mediated increases in R-5-P and PRPP were mitigated. (Yeh et al. 1984) PRPP is used in the salvage pathways for both purine and pyridine nucleotides

These assays were adapted for studies of cultured cells and David Valle showed P5CDH deficiency as the cause of Type 2 hyperprolinemia, (Valle et al. 1974) and the initial characterization of the mechanisms for regulating pyrroline-5-carboxylate reductase (Valle et al. 1975). The recognition that P5C was not only the immediate degradative product of proline but also its direct biosynthetic precursor, a relationship unique in intermediary metabolism, led to a model linking the two substrates in a metabolic cycle catalyzed by PRODH and PYCR (Phang et al. 1980; Yeh et al. 1984). This proposed proline cycle is strategically connected with other major pathways and exhibits biochemical symmetry. Additionally, it can function as a redox shuttle into mitochondria. Dr. Curt Hagedorn provided proof of concept using [^3^H]-glucose and [^14^C]-P5C showing that electrons were transferred from [1-^3^H]-glucose to NADP^+^ and the NADP^3^H was used by P5C reductase to form [^3^H]-proline (Hagedorn and Phang 1983, 1986) **(**Fig. 1**)**. The oxidation of proline in mitochondria passed electrons and ^3^H to form ^3^H_2_O (Hagedorn and Phang 1986). Another support for redox transfer was provided by the transfer of P5C oxidizing equivalents which oxidized NADPH to form a metabolic interlock with the oxidative arm of the pentose phosphate pathway (oxPPP), i.e. G6P dehydrogenase and 6-PG dehydrogenase coupled by NADP^+^/NADPH. Using the formation of ^14^CO_2_ from [1-^14^C]-glucose as an indicator of this interlock (Yeh and Phang 1981), the addition of micromolar concentrations of P5C markedly increased the oxidative PPP (oxPPP). Using normal versus G6PD deficient human erythrocytes, Dr. Grace Yeh showed that not only the OxPPP was activated but also the production of phosphoribosyl pyrophosphate (PRPP) and nucleotides was markedly increased by the addition of P5C (Yeh et al. 1984; Yeh and Phang 1981). These early findings were outlined in a review edited by Earl Stadtman and Bernie Horecker for *Current Topics in Cellular Regulation* (Phang 1985). This was the first model of an enzyme-based regulatory function for proline metabolism and introduced the “proline cycle.”

## Early studies on regulation of PYCR activity

PYCR was studied in extracts of various cultured cells (Valle et al. 1975, 1973) and purified and characterized from human red cells by Drs. Grace Yeh and Marshall Merrill (Merrill et al. 1989). In a line of leukemia cells which were partial auxotrophs for proline, catalytic activity was decreased compared to normal cells, and was not sensitive to feedback inhibition by proline; it also exhibited preference for NADPH over NADH. These findings suggested different isozymes of PYCR, one of which was deficient in the leukemic cell line (Lorans and Phang 1981). Using the yeast complementation strategy, Valle’s lab at Johns Hopkins cloned two genes for the human enzymes and characterized their products (Dougherty et al. 1992). Subsequently a third isozyme was identified, and using the in vitro translated products of the three genes, the specific PYCRs were characterized for their differential preference for reduced pyridine nucleotides, their sensitivity to proline inhibition and their cellular localization (De Ingeniis et al. 2012). Of the three isozymes, designated PYCR 1,2,3 (PYCR3 aka PYCRL), PYCR1 and 2 prefer NADH and are in mitochondria whereas PYCR3 prefers NADPH and is in the cytosol. PYCR2 is sensitive to feedback inhibition by proline. Using Isotope dilution methods, Dr. De Ingeniis suggested that P5C from ornithine was routed to PYCR3 in the cytosol, but additional studies are needed to confirm the compartmentation.

## Proline metabolism and cancer

Although there were metabolomic correlations of proline with cancer, no specific metabolic function was convincingly shown to alter cancer cell behavior. An important discovery was made by Vogelstein’s lab at Johns Hopkins using an adenoviral wild type P53 construct. Expression of P53 rapidly and robustly induced proline dehydrogenase (PRODH), aka proline oxidase, which was earmarked as PIG-6 (P53-induced gene-6) (Polyak et al. 1997). The upregulation of PRODH was thought to provide an anaplerotic source for the tricarboxylic acid cycle. However, cytotoxic treatment to raise P53 levels or doxycycline-initiated ectopic expression of PRODH resulted in apoptosis mediated by ROS (Donald et al. 2001; Hu et al. 2007). Dr. Yongmin Liu showed that inactivating ROS with co-expression of superoxide dismutase 2 mitigated the PRODH effect (Liu et al. 2005). Arousing considerable excitement, these findings suggested that the proline metabolic axis may be involved in cancer (Hu et al. 2007). The complication is that hypoxia can also upregulate expression of PRODH resulting in pro-survival autophagy (Liu et al. 2012a). Whether apoptosis or survival by autophagy depends on the metabolic context, and the effect is not mediated through HIF-1 but through AMPK. By contrast, PRODH activated by hypoxia through AMPK results in prosurvival autophagy (Liu et al. 2012a). This dual function is confusing in that it depends on metabolic and cellular context. In human cancers of the colorectum, stomach, pancreas and kidney, PRODH is downregulated compared to adjacent normal tissues (Liu et al. 2009). Dr. Wei Liu showed that a microRNA (miRNA-23b*) found in kidney tumor cells but not in normal kidney cells, decreases PRODH protein at the level of translation thereby decreasing its proapoptotic effect (Liu et al. 2010).

Recent studies by Dr. Sarah Fendt in Leuven, Belgium showed that breast cancer cells grown as spheroids in 3-D cultures had a dependence on proline and PRODH for growth whereas they did not when grown in 2-D monolayers (Elia et al. 2017). The growth of cells in 3-D spheroids were markedly slowed by treatment with tetrahydrofuroic acid (THFA), an inhibitor of PRODH activity. Thus the functionality of the proline regulatory axis is complicated (Elia et al. 2017). Importantly, these properties of proline metabolism were described with classical allusions as “Janus-like” (Burke et al. 2020).

The PRODH side of the cycle is regulated by P53 (Donald et al. 2001), PPARγ (Pandhare et al. 2006) and AMPK (Liu et al. 2012a). It uses FAD as electron acceptor at site II of the electron transport chain, a feature shared with succinate dehydrogenase (SDH). Additionally, PRODH and SDH activities are inhibited by the other’s substrate, and PRODH and SDH have protein–protein interactions (Hancock et al. 2016). The differential growth-supporting effects of PRODH in monolayer versus 3-D cultures is striking and demonstrate microenvironmentally influenced regulatory effects which are important in the context of metastases.

### Proline biosynthesis

On the biosynthetic side of the cycle, ALDH18A1 codes for P5C synthetase which catalyzes the reaction from glutamate to P5C. The reduction of P5C to proline is mediated by 3 isozymes, PYCR1, PYCR2 AND PYCRL also known as PYCR3. PYCR1 and PYCR2 have 85% sequence homology but PYCR2 is sensitive to proline inhibition whereas PYCR1 is not. Furthermore, PYCR3 is cytosolic and prefers NADPH whereas PYCR1/2 prefer NADH and are located in mitochondria matrix. The central role of the PYCRs is emphasized by recent reviews (Li et al. 2021; Hu 2021; Bogner et al. 2021).

In a collaboration with Dr. Chi Dang and Dr. Teresa Fan, we showed that c-MYC, a widely expressed oncogene, markedly upregulates the expression of ALDH18A1, the gene encoding P5C synthetase and of the genes for all 3 isozymes of P5C reductase (Liu et al. 2012b). Additionally, knockdown of P5C synthase alone or the PYCRs in concert reversed the effect of c-MYC on the growth of tumor cells. It was known that the utilization of glutamine was under c-MYC regulation. Not only the enzymes of proline synthesis were increased with c-MYC but also the flux of ^13^C glutamine to proline. In fact, proline is an important product from glutamine, and the apparent dependence of cancer cells on glutamine may be largely due to this metabolic fate (Grinde et al. 2019). Subsequently, others showed that proline synthesis plays a critical role in numerous cancers, including breast cancer, hepatocellular carcinoma, pancreatic ductal adenocarcinoma, non-small cell lung cancer and others (D'Aniello et al. 2020). These findings as well as tissue culture models clearly show that the proline synthetic axis plays an important role and that it may be the key pathway reprogrammed to enable tumor progression and proliferation.

### Regulation of proline synthesis in cancer

**Fig. 2:**
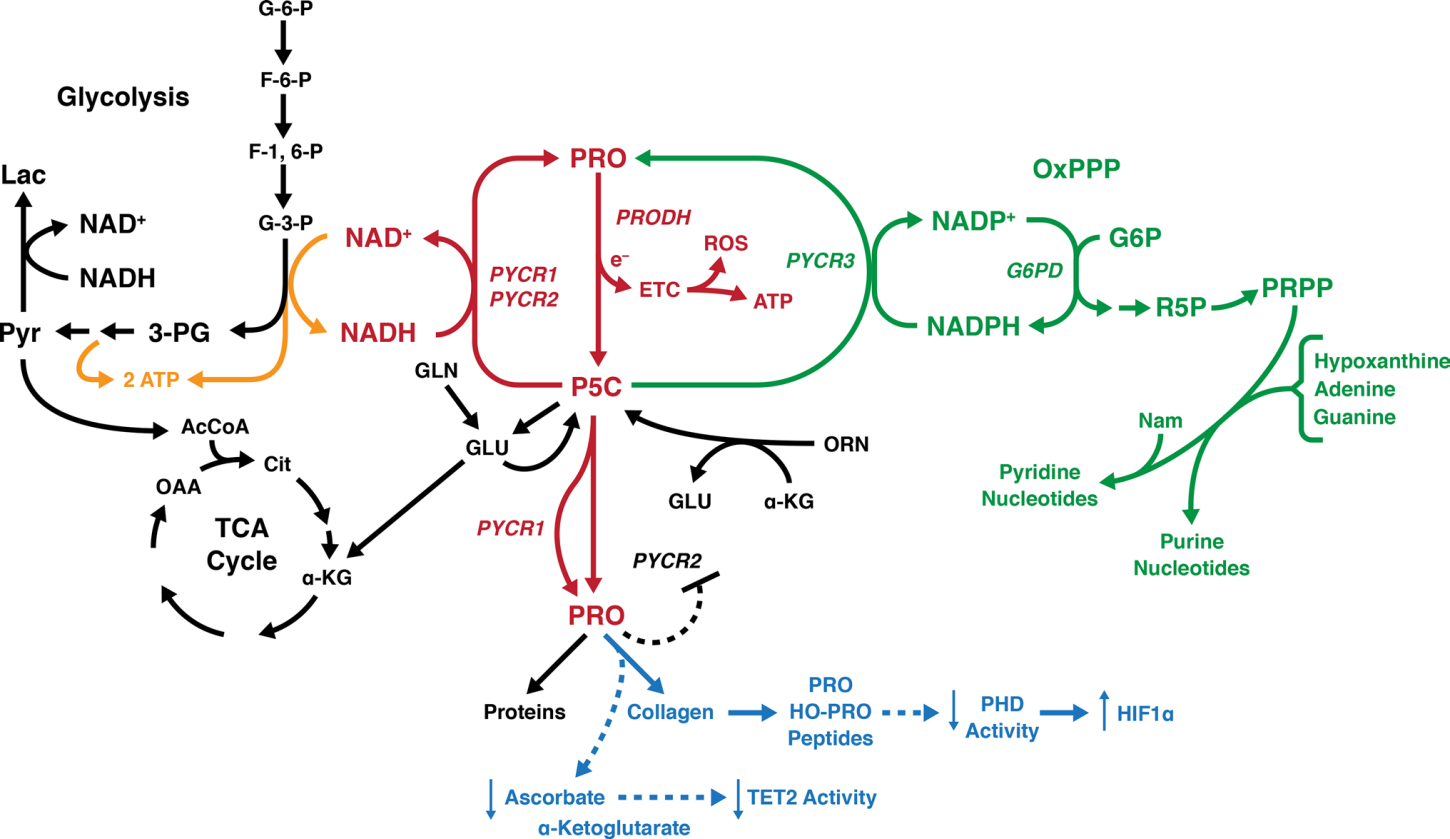
Current regulatory model for “Proline Metabolism and Cancer Reprogramming.” The “Proline-P5C Cycle” is shown in red with proline dehydrogenase transferring electrons from proline to the ETC in mitochondria which can generate ATP or ROS. Shown in green is the interlock between PYCR3 and glucose-6-P Dehydrogenase coupled by NADP^+^/NADPH in the cytosol. This linkage allows P5C to increase the production of PRPP and the generation or maintenance of pyridine and purine nucleotides. Shown in orange, the coupling of PYCR1/2 to the glycolytic pathway and the production of collagen shown in blue. The regeneration of NAD^+^ increases the flux through the glycolytic pathway, obviating the diversion of pyruvate to lactate. Instead, pyruvate is converted to acetyl CoA into the TCA Cycle. The increased synthesis of collagen from biosynthesized proline consumes acorbate and α-ketoglutarate, two substrates necessary for TET2 activity. The degradation of collagen generates prolyl and hydroxyprolyl peptides which are inhibitors of PHDs, aka EglNs, activity which will increase the levels of HIFs. Other regulatory links between PYCR1 and extracellular matrix exist. Their identity and mechanisms have been reviewed (D’Aniello et al. 2020). For intracellular localization of the respective enzymes (Phang 2019)

What are the mechanisms which regulate the level of proline synthetic enzymes and the level of proline synthesis? Since the identification of c-MYC and PI3K-AKT in upregulation of proline synthesis, additional mechanisms have been identified. In neuroblastoma (NB), myeloid zinc finger 1 (MZF1) and MZF1 antisense RNA (MZF1-AS1) are transcriptional regulators of proline synthesis and NB progression (Fang et al. 2019a). Kindlin 2, a widely expressed protein critical for integrin mediated cell-EM adhesion and signaling, interacts with PYCR1, increasing proline synthesis and cell proliferation (Guo et al. 2019). Depletion of Kindlin 2 reduces PYCR1 levels, increases ROS production and abolishes ECM stiffening-induced increase of proline synthesis and cell proliferation. Pinch-1, a cell-extracellular adhesion protein, promotes proline biosynthesis by increasing the expression of P5CS (Cui et al. 2021). Interestingly, SIRT3 regulates proline synthesis by deacetylation of PYCR1 enzyme protein and increasing proline synthesis (Chen et al. 2019), and knockdown of PYCR1 in lung adenocarcinoma inhibits the proliferation and invasion by modulating JAK/STAT (Gao et al. 2020). Inhibitors of HDAC induce PRODH transcription and anti-apoptotic autophagy in triple negative breast cancer (Fang et al. 2019b). (Fig. 2).

### Proline metabolism and nucleotides

Our initial work on the proline cycle included the identification of a metabolic interlock between the reduction of P5C and the oxidative arm of the Pentose Phosphate Pathway, coupled through the NADPH/NADP^+^ redox couple (Phang 1985). In human erythrocytes without mitochondrial enzymes, the addition of low concentrations of P5C increased the flux through the oxPPP, the production of PRPP and the generation of ribonucleotides (Yeh and Phang 1981). The coupling with the rate-limiting enzyme of the oxPPP (G6PD) was shown using red cells from patients with G6PD deficiency in which the P5C effect was absent (Yeh et al. 1984). The linkage of the oxPPP may be especially important during the activation of cell proliferation (Buchanan et al. 1982). A recent publication showed that CRISPR-Cas9 knockdown of mitochondrial NAD Kinase (NADK2), with depletion of NADP^+^ and NADPH in mitochondria made cells auxotrophic for proline (Tran et al. 2021) (Zhu et al. 2021). This was due to the inability to produce P5C from glutamate because P5CS activity has an obligate requirement for NADPH in enzyme assays. NADH mediates little activity. (Wakabayashi and Jones 1983) Cultured lymphoma and lung cancer cells showed depletion of total pyridine nucleotides (both reduced and oxidized) with knockdown of PYCR3 but not knockdown of PYCR1/2 (Liu et al. 2015). Interestingly Tran et al. showed that depletion of NADK2 decreased newly synthesized nucleotides which could be mitigated by the addition of NADK2 or proline. (Tran et al. 2021) The mechanism for this metabolic interlock between proline and nucleotides strongly suggests that the pathways are linked, but the precise mechanism needs additional elucidation. But the metabolic and regulatory interlock between proline synthesis and ribonucleotide and pyridine nucleotide production may be important especially during transition states of proliferation or stress where increased ribonucleotides or pyridine nucleotides are needed. (Fig. 2).

### Proline synthesis and collagen formation and metabolic reprogramming

What is the common denominator for PRODH and P5CS in the context of tumor growth and progression? An obvious commonality is that both PRODH and P5CS are sources of P5C. Even though PRODH is degrading proline, it, like P5CS, produces P5C as substrate for the PYCRs to produce proline. Interestingly, several investigators have suggested that collagen formation is dependent on biosynthesized proline (Hamanaka et al. 2019). Whether this is due to compartmentation or is due to parametabolic effects, e.g. the effect of PYCR3 not only produces proline but also R-5-P and PRPP (shown in Fig. 2 in green) (Phang and Liu 2012), will require additional studies.

The collagens contain proline and its hydroxylated derivative, hydroxyproline, in disproportionately high amounts. In fact, since hydroxyproline is formed after proline peptide linkage into pro-collagen, the total percentage of proline incorporated may be as high as 25% of the total amino acids. In cancer tissue, by the method of differential ribosome codon reading (diricore), quantitating charged and uncharged specific tRNAS attached to ribosomes, uncharged prolyl-tRNA shows that proline is the rate-limiting amino acid (Loayza-Puch et al. 2016). Additionally, biosynthesized proline is preferentially incorporated into collagen and the rate of biosynthesized proline in collagen formation is upregulated by TGF-B_1_ (O'Leary et al. 2020). The further processing of incorporated proline in procollagen requires the action of prolyl hydroxylases (C-P4Hs), a process requiring α-Ketoglutarate Fe^2+^ and Vitamin C. A similar hydroxylation of specific proline residues on HIF protein allows it to be degraded in the presence of oxygen as a sensing mechanism for hypoxia. A similar dioxygenase reaction catalyzed by the TETs and JmjC to demethylate DNA and histones, competing for the same co-substrates as the prolyl hydroxylases has been proposed as the mechanism linking proline metabolism to epigenetics (D’Aniello et al. 2020). Thus, the proline metabolic axis may provide a regulatory connection to link energy metabolism, redox regulation, collagen synthesis, the microenvironment and epigenetics (D’Aniello et al. 2020) (Fig. 2). Although previously investigated in a variety of contexts, the recent association of proline metabolism with collagen synthesis and degradation places new emphasis on prolidase, aka PEPD, the enzyme which catalyzes the final step in collagen degradation. The peptide-linkage formed by a tertiary nitrogen is not hydrolyzed by common proteases or dipeptidases. Instead, the X-PRO or X-HOP bonds require a specific enzyme. Dr. Palka and colleagues in Bialystok, Poland have shown that the release of proline from imidodipeptides can be rate-limiting for collagen synthesis under certain metabolic conditions. Less intuitively obvious, prolidase can bind to TGF-B_1_ and increase the synthesis of PYCR1 (Misiura and Miltyk 2020). A number of activators and inhibitors of prolidase are known, but additional studies are needed to show their clinical effects in cancer.

### Anti-cancer agents targeting the proline regulatory axis

With these recent discoveries of specific molecular mechanisms which may be downstream to the proline regulatory axis and proline cycle, experiments addressing possible inhibitors of the proline cycle elements as well as downstream events are critically important. Potential target sites on PYCR protein have been shown and tested experimentally to identify inhibitors (Tanner et al. 2018; Tanner 2019). Another publication from this group describes “*In crystallo* screening of proline analog inhibitors” and they characterized the inhibitory effect of N-forml-L-proline (NFLP) (Christensen et al. 2020; Bogner et al. 2021). With the discovery of unexpected pathways influenced by the proline regulatory axis, many new pharmacologic approaches may be discovered and tested in monolayer and 3-D cell cultures, in CRISPR knocked down ectopic tumor explants and in induced tumors in animals. A number of agents have already been proposed and tested. Inhibitors and the enzymes they target include: tetrahydrofuroic acid, *N*-propargylglycine (Scott et al. 2021) inhibitors of PRODH; NFLP, inhibitor of PYCR; halofuginone, inhibitor of prolyl t-RNA synthase; budesonide, inhibitor of collagen synthesis. These are published reports of inhibitors of the proline regulated axis and its downstream effectors (D'Aniello et al. 2020). A recent report identified Phenyl-substituted aminomethylene bisphonates as inhibitors of human P5C reductase and antiproliferative activity in tumor cells (Forlani et al. 2021). Hopefully, many others will emerge and future clinical trials especially as adjuncts for chemotherapy drugs may lead to novel therapies.

## Future studies

The validity of the model suggesting that proline metabolism forms a crosstalking hub linking extracellular signaling, energy metabolism, microenvironmental signaling and metabolic epigenetics, may require more specific molecular and spatiotemporal definition. These linkages may not function simultaneously, but may occur in sequence and with regulatory feedback. The “Janus-like” functions of PRODH may require further elucidation of P53 mutations which may have loss or gain of function in a specific tumor. The compartmentation of the various enzymes and more interestingly, the movement of interacting enzymes, such as Kindlin2 and PYCR1 moving Kindlin2 from cytosol to mitochondria, may be critical in the signaling process (Guo et al. 2019). With the establishment of 3 isozymes for PYCR and their apparent compartmentation, it would be important to determine whether the transit of PYCR 1,2 through the cytosol may have transient function in the cytosol before establishing residence in the mitochondrial matrix. As mentioned earlier, the unique tautomeric equilibrium between glutamic semialdehyde, the open-chain form and pyrroline-5-carboxylate, the ring form, may have consequence in compartmentation and in mitochondrial transport. Not only is P5C and its intracellular compartmentation important but the transit of P5C between cells and tissues (Fleming et al. 1989) may be a mechanism of intercellular and interorgan redox exchange. Of special interest is the effect of proline metabolism on stem cell identity (D’Aniello et al. 2015). Additionally, it was recognized years ago that collagen synthesis and degradation may provide a source of intracellular free hydroxyproline as a regulatory metabolite before collagen is excreted (Chojkier et al. 1982). With recent discovery of its regulatory properties, hydroxyproline, itself, produced through collagen may be an important nutritional endpoint (Wu et al. 2019). Finally, the identification of natural metabolites or the synthesis of novel compounds which interact with the proline/P5C enzymes to modulate the pathways critical for metabolic epigenetic reprogramming may provide novel strategies for cancer therapy.

## Summary

This brief perspective traces the discovery of the proline/P5C metabolic cycle with effects on redox and substrate synthesis, but the extensiveness and complexity of proline metabolic signaling was not expected. Now we know that layers of regulation and interlocks connect the proline regulatory axis with many other systems. It is likely that many other physiologic and pathophysiologic systems are modified by mechanisms derived from proline metabolism. The in vitro regulatory effects of MYC on the enzymes of proline synthesis was corroborated by high levels of these enzymes in a variety of clinical cancers, and knockdown of proline synthesis markedly inhibited the growth and progression of cultured tumor cells. Definition of enzyme structures have led to the identification and characterization of specific inhibitors as potential agents in the adjunctive treatment of cancer.
